# sEMG Signal Acquisition Strategy towards Hand FES Control

**DOI:** 10.1155/2018/2350834

**Published:** 2018-03-14

**Authors:** Cinthya Lourdes Toledo-Peral, Josefina Gutiérrez-Martínez, Jorge Airy Mercado-Gutiérrez, Ana Isabel Martín-Vignon-Whaley, Arturo Vera-Hernández, Lorenzo Leija-Salas

**Affiliations:** ^1^División de Investigación en Ingeniería Médica, Instituto Nacional de Rehabilitación “Luis Guillermo Ibarra Ibarra”, Calz. México-Xochimilco No. 289, Col. Arenal de Guadalupe, Tlalpan, 14389 Ciudad de México, Mexico; ^2^Facultad de Ingeniería, Universidad La Salle, Benjamín Franklin 45, Col. Condesa, Cuauhtémoc, 06140 Ciudad de México, Mexico; ^3^LAREMUS, Sección Bioelectrónica, Departamento de Ingeniería Eléctrica, Centro de Investigación y de Estudios Avanzados del Instituto Politécnico Nacional, Av. Instituto Politécnico Nacional 2508, Col. San Pedro Zacatenco, Gustavo A. Madero, 07360 Ciudad de México, Mexico

## Abstract

Due to damage of the nervous system, patients experience impediments in their daily life: severe fatigue, tremor or impaired hand dexterity, hemiparesis, or hemiplegia. Surface electromyography (sEMG) signal analysis is used to identify motion; however, standardization of electrode placement and classification of sEMG patterns are major challenges. This paper describes a technique used to acquire sEMG signals for five hand motion patterns from six able-bodied subjects using an array of recording and stimulation electrodes placed on the forearm and its effects over functional electrical stimulation (FES) and volitional sEMG combinations, in order to eventually control a sEMG-driven FES neuroprosthesis for upper limb rehabilitation. A two-part protocol was performed. First, personalized templates to place eight sEMG bipolar channels were designed; with these data, a universal template, called forearm electrode set (FELT), was built. Second, volitional and evoked movements were recorded during FES application. 95% classification accuracy was achieved using two sessions per movement. With the FELT, it was possible to perform FES and sEMG recordings simultaneously. Also, it was possible to extract the volitional and evoked sEMG from the raw signal, which is highly important for closed-loop FES control.

## 1. Introduction

Neurological disabilities are caused by damage of the nervous system (which includes the brain and spinal cord); this damage results in the loss of capacity to move and manipulate things, especially if fine movements are required [1]. The effects of many neurological conditions can vary greatly from person to person, as well as from time to time for the same person. People with neurological conditions, such as a stroke, may present hand motor impairment and deficit in motor execution, severe fatigue and/or weakness, impaired hand dexterity, tremors, spasticity, abnormal muscle synergies, and deficit in motor planning and motor learning [2]. Stroke survivors may have great difficulty to modulate muscle activation, and their ability to span region is curtailed [3].

Biomedical signals, such as surface electromyography (sEMG), play a significant role in the measurement of the electrical muscle contraction. Plus, its analysis is one of the standard procedures used to identify muscle actions in normal and pathologic conditions. sEMG signals can be used for various applications, which include identifying neuromuscular diseases, controlling signals for orthotic or prosthetic devices [4], anticipating movements of the muscles [5], controlling machines or robots, or detecting hand gestures to improve the quality of life [6].

sEMG patterns during movements exhibit a great deal of intersubject, intermuscle, and context-dependent variability. Understanding the sEMG interactions in hand movements is a challenge [7]. Several researches have been directed to determine the extent to which each muscle participates in each synchronous and time-varying synergies for an individualized human hand motor pattern [8] or to predict the sEMG patterns associated with static hand postures [9]. These studies show the importance of considering different intensities and durations of sEMG bursts, temporal patterns, strength of the muscle contraction [10], and muscle synergy as a framework for sEMG patterns of hand postures.

sEMG patterns are used for neuromuscular biofeedback [11], robot-aided [12] training, and neurorehabilitation, as well as to control devices such as neuroprosthesis based on functional electrical stimulation (FES), to mimic a neuromuscular function for both upper and lower extremities [13], or to enhance hand motor recovery when physical therapy alone is ineffective in stroke patients [14] or with spinal cord injury [15].

Several techniques have been employed for addressing human hand movement patterns from sEMG signal. Techniques, such as an adaptive neuro-fuzzy inference system integrated with a real-time learning scheme and time-frequency features, have been used to identify hand motion commands suitable for hand prosthesis control [16]. Ordinal pattern analysis is used to describe corrections of sEMG recordings during hand open and hand close states. The results suggest that the mutual information analysis has potential in identifying different hand movements [17]. Usually, wavelet transformations and artificial neural network classifiers are used for hand movement analysis [10]. The Hilbert-Huang transform is another technique used to detect, measure, filter, and decompose sEMG signals in order to identify patterns in time, frequency, or space or the combination of flexion/extension arm movements. However, the sEMG patterns can present abnormal muscle synergies and be indistinguishable [18]. This fact could make the classification in some stroke patients more difficult; for example, a solution proposed in [3] is to use voice recognition as an auxiliary in a sEMG-driven actuated glove for clinical therapy purposes.

Recognizing sEMG signals with the aim of controlling assisting devices is not only concerned about feature extraction and classification of signals but the acquisition site is also of major importance.

M-wave is an electrophysiological response evoked by electrostimulation detected in standard sEMG. It has been studied widely in order to verify the functionality of the stimulation site measurement over the target muscle, which closely relates to muscle fiber recruitment. This electrophysiologically driven approach is expected to lead to the identification of selective electrode configurations of an array for functional movements [19]. However, finding the best electrode configuration for sEMG recording to get the right sequence for movement activation still represents a challenge.

This paper is related to the acquisition and analysis of sEMG signals for active movements and to obtaining usable hand patterns with simultaneous placing of recording and stimulation electrodes on the forearm, for the eventual control of a neuroprosthesis to aid in motor neurorehabilitation of patients suffering from a stroke aftermath.

The presented technique is based on an array of recording and stimulation electrodes on the forearm, used to acquire sEMG signals from five hand motion patterns from six able-bodied subjects, and the effects of this technique over functional electrical stimulation (FES) and volitional sEMG combinations.

## 2. Methodology

### 2.1. Identification of sEMG Locations

The first step was to find the best electrode positioning for sEMG recording. This position was found at the belly of the muscle, on the upper part of the forearm, which is formed by the following muscles: brachioradialis, palmaris longus, flexor carpi radialis, flexor carpi ulnaris, extensor carpi radialis longus, and extensor carpi ulnaris. Stimulation is performed at the ends of the same muscles.

In order to make sure that the electrodes were placed on the same positions for the different trials for each subject, a personalized template was made. This template was created as follows: for bipolar channel placement, eight spots, where the electrodes would be placed, were allocated and marked on a piece of acetate paper. Then, the unique physical characteristics of the individual and the positions of five stimulation bipolar electrodes were marked on the same paper. Once the places were allocated and the personalized template was designed, sEMG acquisition was carried out.

### 2.2. sEMG Signals Acquisition

Six able-bodied subjects were included for the acquisition of sEMG signals, their age ranged from 21 to 33 years old, three males and three females. The subject was sitting in a comfortable position with his/her right arm supinated and leaning on the table. The subject was asked to perform an isometric contraction for five movements: hand open, power grasp, fine pinch, pronation, and supination. While contraction was active, the forearm muscles that participate in the motion were palpated and located.

**Figure 1:**
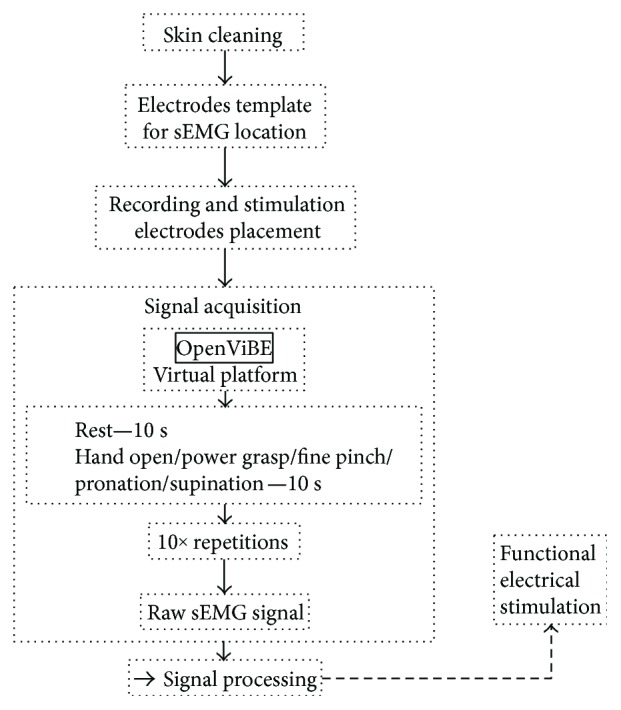
Electrode placement using a personalized template to find sEMG signal for acquisition task and stimulation location. After cleaning the skin and placing the electrodes, the isometric contraction (hand open, power grasp, fine pinch, pronation, and supination) was performed by the subject during 10 seconds, with 10 seconds for rest. The task was repeated 10 times. A session included a task for each movement.

The subject's skin was cleaned using an alcohol swab in order to reduce impedance and have a better coupling for the skin-electrode interface. Afterwards, the template was placed on the subject's forearm and marked; these were the spots where the electrodes should be placed.  Figure 1 shows this procedure. The electrodes were kept in contact with the skin with a tubular mesh; this also reduced artifacts due to cable movements.

**Figure 2:**
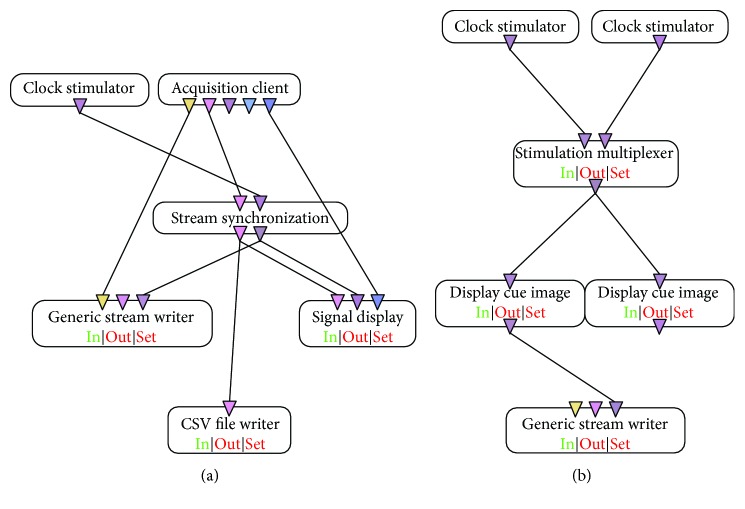
OpenViBE flow diagram used to acquire raw sEMG signal (a); image cue synchronization control (b). This algorithm completes a movement task.

Electrodes were connected to an open-source platform called OpenViBE. This acquired the sEMG signal through a compatible open hardware acquisition device (OpenBCI) which was connected to a designer space, where an algorithm was designed for trial tasks (Figure 2).

OpenViBE configuration was 24 for gain, 250 Hz for sampling rate, and eight channels for sEMG. The subject was asked to perform the movement shown in a cue image while it was on the screen. The task started with a rest of 10 seconds, and it continued with a ten-second isometric contraction of hand open, power grasp, fine pinch, pronation, or supination, depending on the trial. Cue images were shown alternatively until the subject completed ten repetitions of the motion. A session was considered completed when two movement tasks were finished (Figure 1). All subjects completed two sessions for each of the mentioned motions. The tasks of sEMG recordings were saved as .csv files that included the information of eight channels and a time vector.

From all the personalized positions, which were based on common regions found for each subject, a universal template that kept the array for recording and stimulation electrodes was designed. It was called forearm electrode set (FELT).

### 2.3. Preprocessing, Selection, and Feature Extraction

**Figure 3:**
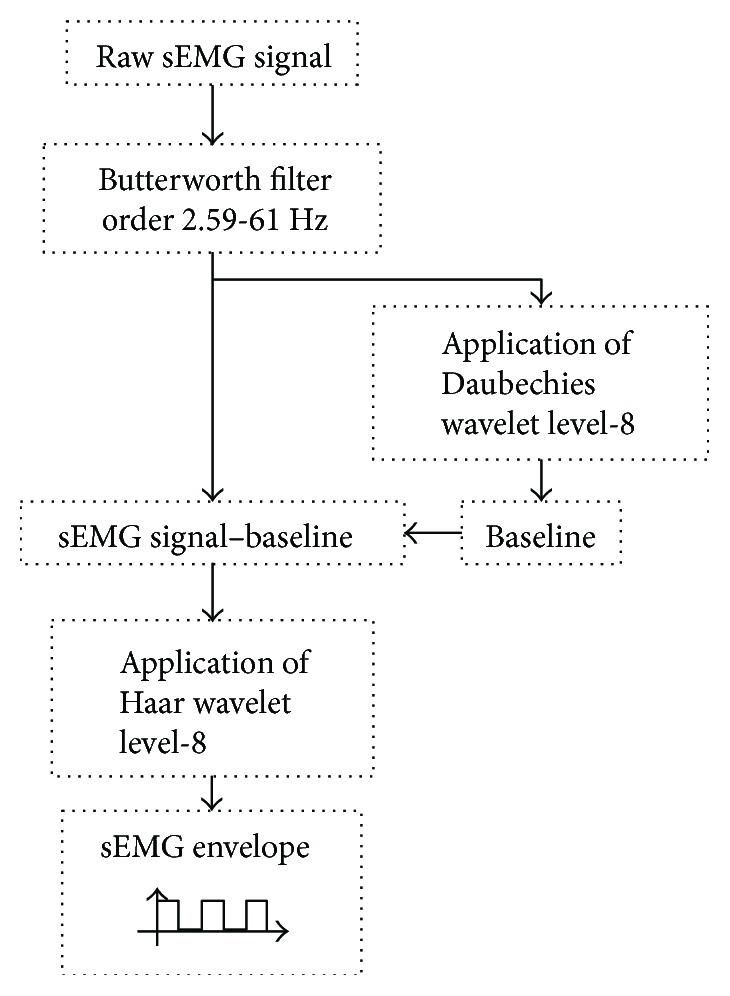
sEMG signal processing algorithm. The signal was filtered for 60 Hz, baseline was subtracted through DWT, and the envelope signal that selected the active pattern was obtained.

Each sEMG record was imported into MATLAB® environment for processing. From the .csv files, information of eight channels and a time vector was extracted. As seen in Figure 3, the signal was cleaned from line interference at 60 Hz by using a Butterworth filter, order 2, with a 59 to 61 Hz bandwidth.

After acquisition, data were conditioned using discrete wavelet transforms (DWT). An eight-level decomposition using mother wavelet Daubechies-4 was applied, and the reconstructed signal was subtracted in order to eliminate baseline drift [20]; this was equivalent to filter a 0.7 Hz signal.

Then, the DWT was applied, again, to an eight-level decomposition, but this time a mother wavelet Haar was used in order to find the envelope of the signal, which was obtained from its reconstruction. This envelope was used to find the parts of the sEMG signal that represented a movement, in this case open hand or power grasp; then it was converted to a logic signal (Figure 3).

In order to find the characteristic features of the five target movements, the following parameters were calculated: mean absolute value—MAV (1), wave length—WL (2), zero crossing—ZC (3), standard deviation—SD (4), integral of absolute value—IAV (5), variance—V (6), and slope sign change—SSC (7).

(1) $$\begin{array}{c} MAV=\frac{1}{n}\overset{n}{\underset{i=1}{\sum }}\left|x_{i}\right|, \end{array}$$

(2) $$\begin{array}{c} WL=\overset{n}{\underset{i=1}{\sum }}\left|x_{i}-x_{i-1}\right|, \end{array}$$

(3) $$\begin{array}{c} ZC=\overset{n-1}{\underset{i=1}{\sum }}\left\{\begin{array}{ll} 1, & x_{i+1}<0,x_{i}>0,\\ 1, & x_{i+1}>0,x_{i}<0,\\ 0, & \end{array}\right. \end{array}$$

(4) $$\begin{array}{c} SD=\sqrt{\frac{1}{n-1}\overset{n}{\underset{i=1}{\sum }}{\left(x_{i}-\bar{x}\right)}^{2}}, \end{array}$$

(5) $$\begin{array}{c} IAV=\overset{n}{\underset{i=1}{\sum }}x_{i}, \end{array}$$

(6) $$\begin{array}{c} V=\frac{1}{n-1}\overset{n}{\underset{i=1}{\sum }}{\left(x_{i}-\bar{x}\right)}^{2}, \end{array}$$

(7) $$\begin{array}{c} SSC=\left\{\begin{array}{ll} 1, & x_{i}>x_{i+1},x_{i}>x_{i-1},\\ 1, & x_{i}<x_{i+1},x_{i}>x_{i-1},\\ 0, & else. \end{array}\right. \end{array}$$

From these parameters, a subset was selected for classification based on separability between movements and classification accuracy. The new set of parameters were used for classification.

### 2.4. Classification

For the classification of sEMG signals, feature and window length analysis were performed for the eight channels. The sEMG envelope signal was used for selection of active patterns at the processing stage. From this ~10 s of sEMG activity, windows of 20 ms, 50 ms, 100 ms, 300 ms, and 500 ms and 1 s and 3 s length, with a 25% overlap, were used to calculate the seven features described in (1), (2), (3), (4), (5), (6), and (7).

A linear discriminant analysis (LDA) was executed for sets of two movements following the process described ahead, in this case for hand open and power grasp:

1. For each subject and each analysis window value, the seven features were extracted for the eight channels; for hand open task and power grasp task.
2. The resulting 56 features obtained from each window were considered as a single trial for each movement.
3. All available trials from the first session (one task per movement) of all subjects were concatenated movement-wise and randomized afterwards.
4. Label classes for each trial were set as 1 for hand open and 2 for power grasp.
5. For each window length value, the analysis was performed ten times.
6. All trials were divided in 70% for a training set and 30% for a testing set.
7. A LDA classifier was trained with the training set.
8. The trials on the testing set were classified with the LDA classifier, and its classification accuracy was calculated as the ratio of correctly classified trials versus the total number of trials.

All subjects' data from the first session (combinations of features, channels and window lengths) that obtained a classification accuracy higher than 90% were chosen as the subset of features used to train the final LDA classifier. Data from the second session, which consisted of hand open and power grasp for each subject, was processed in the same way and was used to test the LDA classifier.

### 2.5. sEMG Recording and FES Application: Acquisition and Processing

For the trials of sEMG signal acquisition during FES application, the acquisition was performed using the OpenViBE platform and OpenBCI device with the same configuration mentioned above. For FES application, a RehaStim 2 electrical stimulator (Hasomed Gmbh, Germany) was used and programmed in an interface developed in Simulink®/MATLAB Environment.

Three able-bodied subjects out of the six that performed the previous trials without FES, age range from 22 to 34 years old, two males and one female, were included for sEMG acquisition. Their skin was cleaned with alcohol and the FELT was placed accordingly. For each target motion, there was a pair of self-adhesive stimulation electrodes (Axxelgard, USA) placed within the FELT, positioned as presented in Table 1.

**Figure 4:**
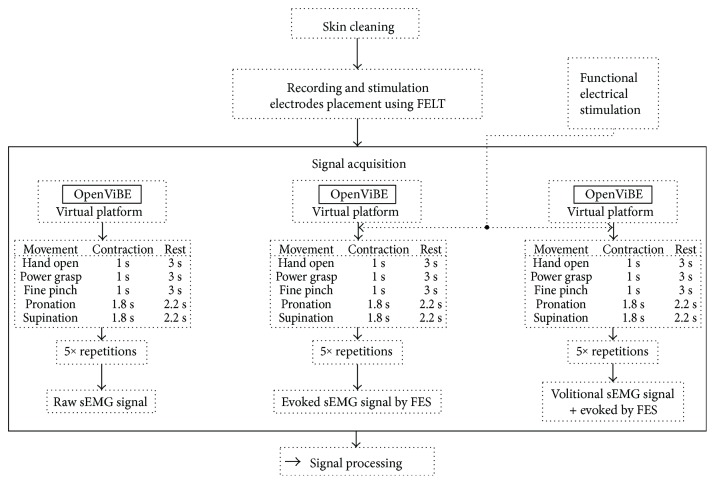
sEMG signal acquisition for tasks (hand open, power grasp, fine pinch, pronation, and supination) with FES stimulation. An isometric contraction was performed by the subject for each part of the trial. The motion was repeated 5 times per part. A session included 5 repetitions of volitional contraction, followed by 5 repetitions of sEMG evoked by FES, and finally, 5 repetitions of volitional contraction plus the evoked sEMG by the FES stimulation.

The subject was asked to perform an isometric contraction for five movements: hand open, power grasp, fine pinch, pronation, and supination, but this time the trial consisted of three parts (Figure 4):

1. Five isometric contractions of the selected movement, each lasting one second with three seconds rest (except pronation and supination: 1.8 active to 2.2 second rest)
2. Five FES stimulations of the selected movement, each lasting one second with three seconds rest
3. Five isometric contractions during FES stimulations of the selected movement, each lasting one second with three seconds rest

The algorithm in Figure 4 was performed once for each movement and subject. The stimulation parameters changed for each movement according to Table 2.

The new records were analyzed for processing the sEMG data because these signals included evoked and/or volitional sEMG as well as the FES stimulus. In order to extract the sEMG evoked/volitional sEMG from the stimulus artifact, a comb-type filter was applied to eliminate the 30 or 50 Hz signal of the stimulus, by means of a Butterworth filter, order two, with a 29 to 31 Hz or 49 to 51 Hz bandwidths, accordingly. All data processing is designed and performed in MATLAB environment. The parameters calculated for these signals are MAV (1) and root mean square (RMS) (8) to compare sEMG of evoked and volitional and evoked signals.

(8) $$\begin{array}{c} RMS=\sqrt{\frac{1}{n}\overset{n}{\underset{i=1}{\sum }}{\left(x_{i}\right)}^{2}}. \end{array}$$

## 3. Results and Discussion

A personalized template was designed for each subject. These templates were used to successfully locate muscle sites and place electrodes for the second trial, with the advantage of a tenfold reduction in location time, approximately. Then, the FELT was designed as a universal array from all the individual templates.

The main purpose of the FELT was to simplify recording and stimulation electrode placing, for a future FES-based neuroprosthesis clinical application for stroke aftermath rehabilitation at upper limb and hand. There are not standardized designs for sEMG recording and FES application. The sEMG signals acquired for open hand and power grasp were used to evaluate the right position of the recording electrodes at the FELT.

One of the objectives of this work was to allocate all electrodes keeping the balance between having available positions to acquire eight sEMG channels and enough place for five bipolar stimulation channels. It is important to mention that since the forearm is a small area, it was difficult to find the right allocation for all the electrodes (stimulation electrodes are 5×5 cm and recording electrodes are 1 cm in diameter) and still have useful signals that could be processed and classified.

Due to this critical disposition, the electrode locations from the personalized templates were assessed through the sEMG signals obtained by means of signal processing and classification of movements.

**Figure 5:**
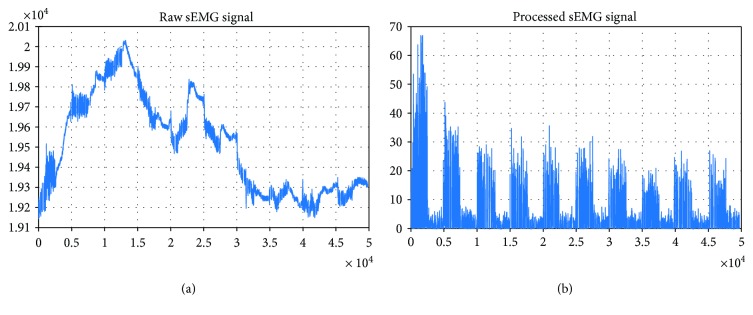
Subject 1, open hand/rest. Comparison of sEMG signal before and after processing using DWT. (a) Raw sEMG signal containing baseline drift and 60 Hz noise. (b) Processed sEMG signal drift-free and visible active and rest patterns.

A baseline drift-free signal was obtained from the raw sEMG signal during the preprocessing stage (Figure 5). All sessions from the six subjects were put through this processing. sEMG signal in Figure 5(a) has a large baseline, while Figure 5(b) shows a cleaner sEMG signal despite original baseline drifting; also, the differences between each contraction repetition are clearer.

The preprocessing analysis and processing method showed that no matter the 60 Hz noise and drifting baseline, the signal could be isolated for feature extraction and classification. It is important to mention that if the acquisition signal was less contaminated, this process could be faster and closer to real time for control applications, which emphasizes the need to design and build a specialized acquisition stage in order to start with the best version of a raw sEMG signal (which can also consider a configuration that allows the simultaneous application of FES, for volitional sEMG extraction).

**Figure 6:**
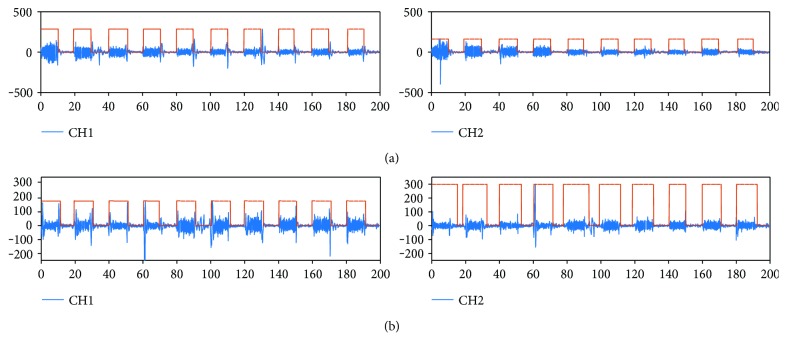
For subject 1, (a) open hand and (b) power grasp, sEMG processed and envelope signal obtained for active pattern selection. Example for channels 1 and 2 of 8.

Figure 6 shows an example of two of the eight sEMG channels processed and the envelope signal obtained, which shows the active sEMG sections selected. These correspond to open hand and power grasp movements.

**Figure 7:**
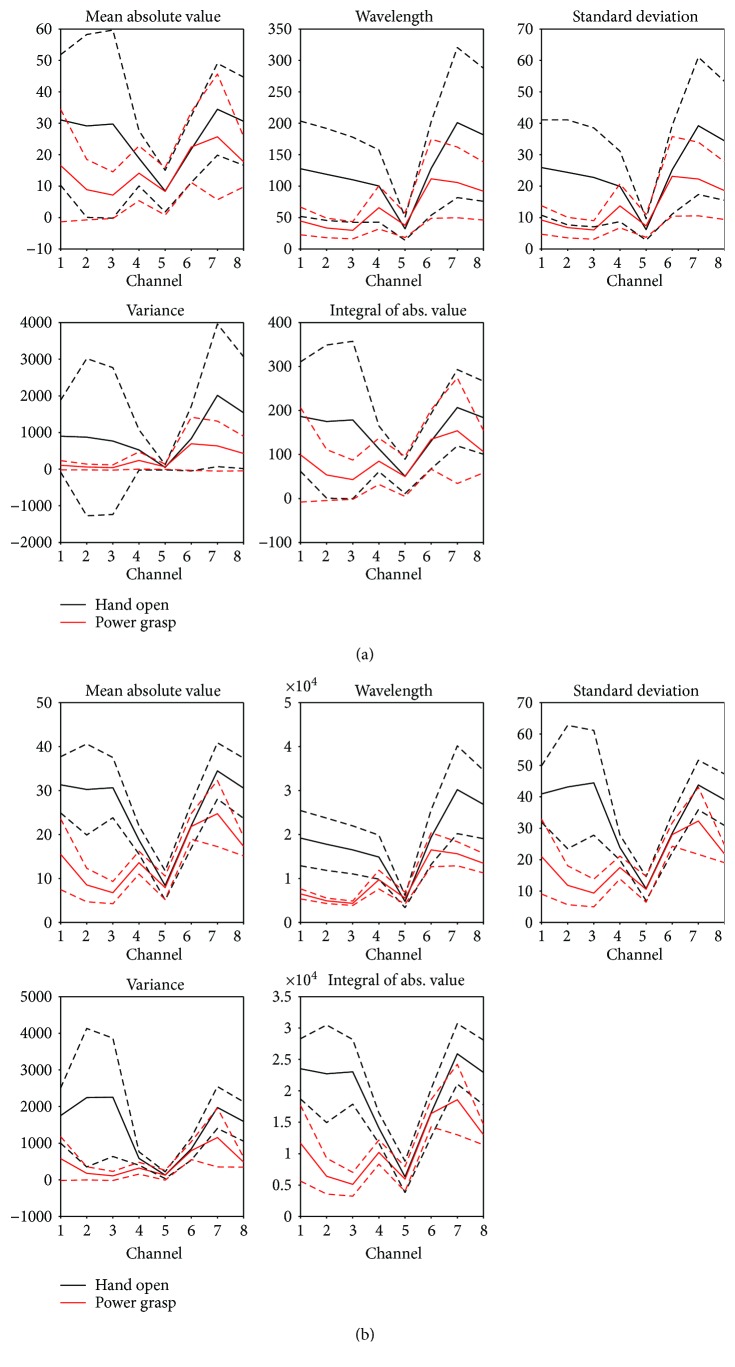
Analysis of window length for (a) 20 ms and (b) 3 s for all features (MAV, WL, SD, IAV, and V) and 8 channels, using data from the 6 subjects.

From the analysis of the combinations of features, channel, and window length for all subjects, it was found that only 5 features (MAV, WL, SD, IAV, and V) yield enough information for classification, above 90% accuracy (Figure 7). In Figure 7(b), it can be observed that when the length of the window was larger, for features like MAV or SD, it was easier to find a clear separation of the value of the parameters. Even the smaller windows, i.e., 20 ms, (Figure 7(a)) performed with an accuracy of 80.69%. Then, it is important to find a compromise between window length and classifier performance.

From this analysis, using 9 out of 10 repetitions of each movement per session and considering session 1 for training and session 2 for testing, it was found that MAV, WL, and SD features and a 0.50 seconds' window length were the best combinations for the classifier to perform with only 4 channels (CH1, CH2, CH3, and CH7) at a 95.83% classification accuracy. The results from all combinations can be seen in Table 3.

This study and analysis was performed to minimize inputs for the classifier, with the aim of getting a closer approach to a real-time application. This analysis is a classification method for multisubjects, used to generate a sEMG-driven control for a FES neuroprosthesis application.

**Figure 8:**
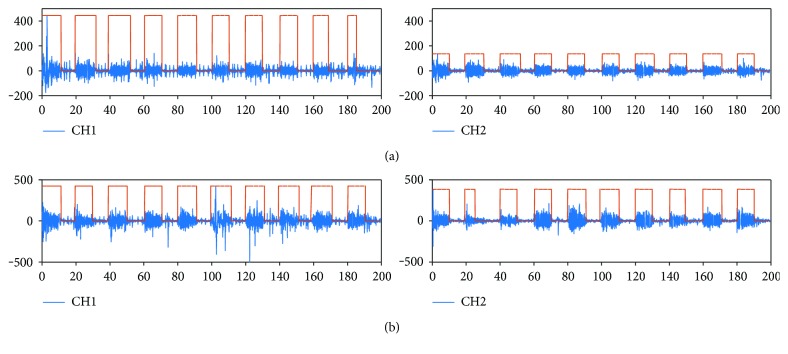
Subject 1 using FELT: (a) channels 1 and 2 for open hand and (b) channels 1 and 2 for power grasp.

An example of the sEMG signals obtained for subject 1 using FELT, for channels 1 and 2, is shown in Figure 8. It can be observed that even though the signal was noisier for this session, the processing algorithm was still able to find the active sEMG sections.

**Figure 9:**
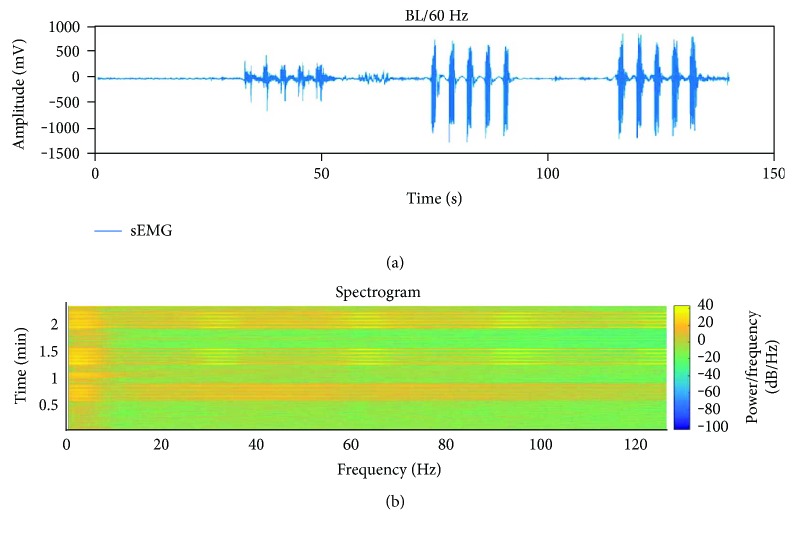
(a) Power grasp sEMG signal recorded from trial (algorithm Figure 4), channel 1. Baseline has been eliminated using algorithm of Figure 2. (b) Spectogram of sEMG signal, where activity in the 30 Hz band for the 2nd and 3rd sets of motions and their harmonics can be observed.

Figure 9 shows the signal resulting from the sEMG (evoked/volitional) and FES stimulus signal acquisition using the FELT.

**Figure 10:**
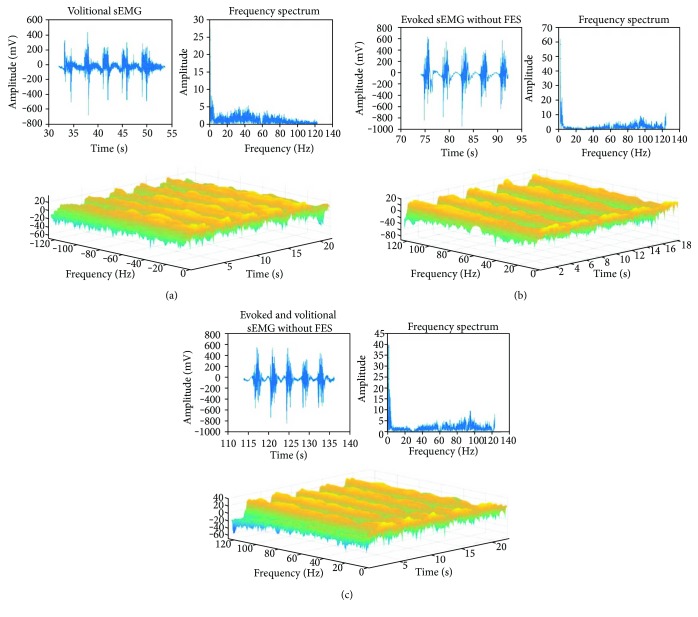
Power grasp, subject 1, channel 1, sEMG signals of the 3 parts of the trial. (a) Set of 5 isometric contractions of the selected movement, each lasting 1 second with 3 seconds rest. (b) 5 FES stimulations of the selected movement, each lasting 1 second with 3 seconds rest. (c) 5 isometric contractions during FES stimulations of the selected movement, each lasting 1 second with 3 seconds rest.

Figure 10 shows each set of repetitions of the 3 parts of the trial. The frequency spectrum and a 3D spectrogram are presented.

**Figure 11:**
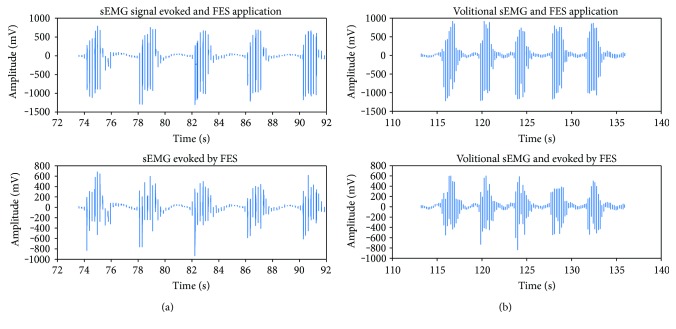
Comparison of sEMG signals between 2 parts of the trial involving FES application. (a) Raw signal including FES (top) and sEMG signal evoked by FES free of the stimulus (bottom). (b) Raw signal including volitional sEMG and FES (top) and volitional sEMG signal and evoked by FES free of the stimulus (bottom).

Figure 11 shows the sets of contractions for sEMG evoked by FES and those from a volitional sEMG contribution used in order to compare the effects of both conditions.

The RMS and MAV values for each repetition were calculated; Table 4 shows an example of these values.

## 4. Conclusions

The design of a personalized template presented in this paper replicates the sEMG signal between sessions. Also, the forearm electrode set (FELT) resulted from the need to find the correct place for eight sEMG bipolar channels and five bipolar stimulation channels (larger electrodes, 5×5 cm) in the forearm, which is a small area for so many electrodes (a total of 27).

Signal processing yielded a very clean signal that preserved sEMG components by using DWT and allowed to differentiate between movements through feature extraction and classification.

We found an optimal combination between window length and number of channels and features, at 0.5 seconds, with four channels and three features (MAV, WL, and SD), which allowed a more efficient classification in terms of time and channels.

The stimulation parameters were selected in order to generate a complete movement without subject discomfort; however, range of movement is yet to be evaluated. As for signal processing, knowing the stimulus frequency beforehand allows the use of a filtering technique feasible for offline and online application. From Figure 10, it is evident that a natural sEMG contraction activates the slow fibers of the muscle, but in the cases of FES application (Figures 10(b) and 10(c)), the fast twitch fibers have a larger contribution to the sEMG record. Additionally, the evoked and volitional sEMG with FES were similar; however, it should be considered that the sample was small and that all subjects were able-bodied. Therefore, a protocol with a bigger sample is needed and it still remains to be seen if these results hold for patients.

Using the FELT, it was possible to perform sEMG recording and FES simultaneously. Moreover, it was possible to extract the volitional and evoked sEMG from the raw signal, which was accomplished without blanking the signal allowing better control techniques to be implemented. This is highly important for closed-loop FES control.

In the evoked/volitional sEMG and FES trials, the FES stimulus was successfully eliminated from the recorded signal leaving a usable sEMG signal for FES control and other applications as orthosis, prosthetics, neuroprosthesis, and other rehabilitation and assistive devices.

## Figures and Tables

**Table 1:** Stimulation electrode positions for each of the five target movements.

Target movement	Electrode position
Power grasp	Finger and wrist flexors. Flexor carpi radialis, flexor carpi ulnaris, flexor digitorum superficialis.
Lumbrical grip	Ulnar nerve. Flexor pollicis longus, flexor digitorum superficialis.
Hand open	Finger and wrist extensors. Extensor carpi radialis. Extensor digitorum.
Pronation	Pronator teres.
Supination	Supinator.

**Table 2:** Stimulation parameters for each subject and target movement.

Subject	Movement	Pulse amplitude (mA_p_)	Pulse width (*μ* s)	Pulse frequency (Hz)	On/Off time (s)
**1**	PG	10	300	30	1/3
	LG	10	300	30	1/3
	HO	10	500	30	1/3
	SU	10	500	50	1.8/2.2
	PR	10	500	30	1.8/2.2

**2**	PG	10	300	30	1/3
	LG	12	300	30	1/3
	HO	10	300	30	1/3
	SU	10	300	50	1.8/2.2
	PR	10	300	30	1.8/2.2

**3**	PG	10	300	30	1/3
	LG	8	400	30	1/3
	HO	12	300	30	1/3
	SU	10	500	50	1.8/2.2
	PR	10	500	30	1.8/2.2

PG: power grasp; HO: hand open; SU: forearm supination; PR: forearm pronation; LG: lumbrical grip, applied through the RehaStim 2 electrical stimulator.

**Table 3:** Analysis of the combinations of selected channels and features with best performance during training, for each window length.

Window length (s)	Channels	Features	Classifier accuracy (%)
0.02	1–3	WL, SD	80.69
0.05	1–3	WL, SD	88.23
0.10	1–3	WL, SD, V	91.56
0.30	1–3, 7-8	MAV, WL, SD, V	93.86
**0.50**	**1–3**, **7**	**MAV**, **WL**, **SD**	**95.83**
1.00	1–4, 7-8	MAV, WL, SD, V, IAV	94.68
**3.00**	**1–3**, **7-8**	**MAV**, **WL**, **SD**, **IAV**	**95.14**

The bold rows correspond to classification accuracies above 95%.

**Table 4:** RMS and MAV values obtained for 3 able-bodied subjects, comparison between sEMG evoked by FES and the combination of volitional and evoked by FES signals. Values obtained from motion of power grasp, channel 1.

Subject	Gender	sEMG evoked by FES		Volitional sEMG + sEMG evoked by FES	
		RMS (mV)	MAV (mV)	RMS (mV)	MAV (mV)
1	Male	147.5061	105.6109	147.4792	104.6412
2	Male	159.2150	109.0613	169.7005	126.7346
3	Female	306.5072	200.5491	215.4075	138.3950
